# Using digital technology as a platform to strengthen the continuum of care at community level for maternal, child and adolescent health in Tanzania: introducing the Afya-Tek program

**DOI:** 10.1186/s12913-024-11302-7

**Published:** 2024-07-30

**Authors:** Angel Dillip, Gloria Kahamba, Richard Sambaiga, Elizabeth Shekalaghe, Ntuli Kapologwe, Erick Kitali, James Tumaini Kengia, Tumaini Haonga, Simon Nzilibili, Mark Tanda, Yasini Haroun, Rachel Hofmann, Rebecca Litner, Riccardo Lampariello, Suleiman Kimatta, Sosthenes Ketende, Johanitha James, Khadija Fumbwe, Fatma Mahmoud, Oscar Lugumamu, Christina Gabunda, Ally Salim, Megan Allen, Eden Mathew, Melania Nkaka, Jafary Liana, Toby Norman, Romuald Mbwasi, Nandini Sarkar

**Affiliations:** 1 Apotheker Health Access Initiative, Box 70022, Dar Es Salaam, Tanzania; 2 D-Tree International, Dar Es Salaam, Tanzania; 3 Sociology and Anthropology Department, University of Dar Es Salaam, Dar Es Salaam, Tanzania; 4 Pharmacy Council of Tanzania, Dodoma, Tanzania; 5 President’s Office Regional Administration and Local Government, Dodoma, Tanzania; 6 Ministry of Health, Dodoma, Tanzania; 7 Inspired Ideas, Dar Es Salaam, Tanzania; 8 Simprints, Cambridge, UK; 9 Institute for Tropical Medicine, Antwerp, Belgium

**Keywords:** Afya-Tek, CHW, ADDO, Health facility, Maternal health, Child health, Adolescent health, Digital health, Community, Primary healthcare

## Abstract

Inadequate care within Tanzania’s primary health system contributes to thousands of preventable maternal and child deaths, and unwanted pregnancies each year. A key contributor is lack of coordination between three primary healthcare actors: public sector Community Health Workers (CHWs) and health facilities, and private sector Accredited Drug Dispensing Outlets (ADDOs). The Afya-Tek program aims to improve the continuity of care amongst these actors in Kibaha district, through a mobile-application based digital referral system that focuses on improving maternal, child and adolescent health. The digital system called Afya-Tek was co-created with users and beneficiaries, and utilises open-source technology in-line with existing government systems. The system guides healthcare actors with individualised decision support during client visits and recommends accurate next steps (education, treatment, or referral). From July 2020 to June 2023, a total of 241,000 individuals were enrolled in the Afya-Tek program covering 7,557 pregnant women, 6,582 postpartum women, 45,900 children, and 25,700 adolescents. CHWs have conducted a total of 626,000 home visits to provide health services, including screening clients for danger signs. This has resulted in 38,100 referrals to health facilities and 24,300 linkages to ADDOs. At the ADDO level, 48,552 clients self-presented; 33% of children with pneumonia symptoms received Amoxicillin; 34% of children with diarrhoea symptoms received ORS and zinc; and 4,203 referrals were made to nearest health facilities. Adolescents preferred services at ADDOs as a result of increased perceived privacy and confidentiality. In total, 89% of all referrals were attended by health facilities. As the first digital health program in Tanzania to demonstrate the linkage among public and private sector primary healthcare actors, Afya-Tek holds promise to improve maternal, child and adolescent health as well as for scale-up and sustainability, through incorporation of other disease conditions and integration with government’s Unified Community System (UCS).

**Supplementary Information:**

The online version contains supplementary material available at 10.1186/s12913-024-11302-7.

## What is already known on this topic

Lack of strengthened continuum of care at community level for maternal, child and adolescent in Tanzania is partly due to fragmented service provision at the primary healthcare level; lack of guidance to aid decision-making processes; and limited use of data.

## What this study adds

The Afya-Tek program showcases an innovative digitally enabled system which strengthens the linkages between private sector Accredited Drug Dispensing Outlets (ADDOs) and public sector community health workers and health facilities. The Afya-Tek program improves the continuum of care at community level and prompt access to care for mothers and children through digital decision support tools, patient referral and tracking. The involvement of the private sector ADDOs shows promise in improving adolescent health due to delivering private, confidential and client centred services.

## How this study might affect research, practice or policy

Afya-Tek’s participatory approach in co-creation of the digital system and implementation processes has been critical to ensure system usability and ownership on the ground. As the first digital health system in Tanzania to link public and private sectors, it works within a framework of Public Private Partnership and contributes to effective linkage, for a scaled-up improvement and sustainability of primary healthcare.

## Introduction

Investing in primary health care (PHC) continues to play an important role in public health needs globally in the stride towards universal health coverage, with estimates suggesting that PHC can address 80%—90% of a person’s health needs throughout his or her life [1]. However, the full potential of primary health systems remains to be realised in most low- and middle- income countries (LMICs) [2]. Primary Health Care is the universal health care which allows for the full participation of community members in implementation and decision making [3]. One aspect of the unrealized potential of PHC is the global challenge of fragmentation within health systems [4]. Central to these challenges is the issue of integration and coordination of care among various health system actors and programs—a goal made especially elusive in LMICs, where program funding typically comes from a variety of sources. Countries continue to face internal competition among various health services for access to finite resources, which is made even more difficult by the prevalence of siloed workforces and health programs [5].

The fallout of these systemic challenges often lies in health outcomes. According to the 2022 Tanzania Demographic and Health Survey (TDHS-MIS), the under-5 mortality rate in Tanzania is still almost double the target set out by the SDGs, currently at 47 per 1000 live births [6]. The maternal mortality rate is similarly more than 3 times higher than the SDG target value, the maternal mortality ratio (MMR) for Tanzania is 104 maternal deaths per 100,000 live births for the seven-year period before the survey [6]; adolescent fertility rate is still high at around 123.44 births per 1000 [7]. Estimates highlight that up to 67% of new-born deaths in sub-Saharan Africa could be prevented through a functional referral system and continuum of care, saving 700,477 lives each year [8]; while other studies showcase that further work needs to be done in Tanzania across the continuum of care for maternal, newborn and child health to improve these outcomes [9].

Tanzania, a low-income East African country, experiences similar barriers to coordination of care at the primary care level. Currently, there are inadequate linkages among the three main primary health system actors: namely, Community Health Workers (CHWs), Accredited Drug Dispensing Outlets (ADDOs), and primary health facilities (HF) [10]. Primary health facilities encompass the decentralised levels of operation of the healthcare system mostly found at village, ward and district levels. They include dispensaries, health centres and district hospitals. While CHWs and HFs are part of Tanzania’s decentralised health system, ADDOs are privately owned community-based drug shops. ADDOs are regulated and under the mandate of the Tanzania Pharmacy Council, yet smaller than pharmacies, and are allowed to dispense over the counter medication and a limited list of prescription medicines [11, 12].

Although there are continuous improvements in infrastructure at the PHC level, there is still limited access to HFs for many people, with large distances to HFs remaining a significant barrier to care-seeking [10, 13]. While CHWs refer clients to HFs for certain conditions, tracking these referrals is difficult and follow-up is limited [10]. ADDOs, which are otherwise able to provide easily accessible medications for certain illnesses or family planning services right within the community, have no formal interaction with CHWs. Additionally, ADDOs have a weak link and inefficient referral system to coordinate client care with the HFs [10]. While there is an official paper-based referral system in place at the PHC level, maintaining and tracking these referrals remains a challenge; HFs are often understaffed and the health workers themselves are overburdened [10, 14–16].

Thus, based on this evidence and the Tanzanian context, it is likely that more formalised linkages among CHWs, ADDOs and primary HFs in Tanzania would strengthen care coordination and increase the potential to improve maternal, new-born and child health outcomes. Given the need to optimise and improve these formalised linkages [10], one potential solution is through digital health innovation [17]. Digital technology allows for opportunities to support universal access to high quality healthcare, as well as strengthen and scale up health promotion, diagnosis, and management; though efforts need to also be made to ensure equitable impact [18, 19]. Additionally, the specific role of country-owned stewardship of digital health and a country’s capacity to achieve this, showcases the potential of Tanzania—given its forwardness in this realm—to optimise on such digital solutions and propel its health systems into the future [20–23].

Thus, we present *Afya-Tek*: a comprehensive digitally-enabled primary health care program in Kibaha, Tanzania. The program utilises unique (system-level) identification of patients via digital technology, as a tool to strengthen and coordinate the three main Tanzania primary health system actors: CHWs, ADDOs, and primary health facilities. The Afya-Tek program provides a unique model of service provision which links both public and private health sector actors at the PHC level, thereby highlighting a promising example of public private partnerships (PPP).

This paper outlines the overall Afya-Tek program, and presents the initial program set up; the various methodologies and components of the Afya-Tek program and system design; as well as preliminary findings and analyses from various lessons learnt and challenges faced. This is then followed by a brief discussion on the program and reflections on ways forward.

### Program set up

#### Consortium partners

The Afya-Tek team is made up of a diverse consortium of partners: *Apotheker Health Access Initiative*, a Tanzanian organisation, leads the program and is responsible for Afya-Tek implementation, sensitization and government engagement; together with *D-tree International—Tanzania*, which is responsible for health system strengthening, including Afya-Tek digital system design, development, capacity building, and monitoring. *Simprints*, a UK-based biometrics company, is responsible for contributing and maintaining the biometric fingerprint scanning technology. The *Institute of Tropical Medicine (ITM)* in Antwerp, Belgium, is tasked with evaluating program activities and outcomes through a Realist Evaluation in order to adapt the program to changing needs, as well as capturing transversal learnings. Another research partner is the *University of Dar es Salaam*, working closely with ITM to support program evaluation activities. Additionally, *Inspired Ideas* is a Tanzanian health-tech start-up, working on a pilot sub-project within Afya-Tek, specifically related to testing feasibility of Artificial Intelligence in select ADDOs. Finally, the program works hand in hand and collaborates with the Tanzanian Government, in particular the Ministry of Health (MoH), the President’s Office—Regional Administration and Local Government (PO-RALG), and the Pharmacy Council Tanzania.

## Methodology

The Afya-Tek program is grounded within the human-centred design process and the Realist Evaluation methodology. The program employed a Realist Evaluation approach that combines quantitative and qualitative research techniques to monitor, intervene and evaluate the program’s impact on health and document learnings around the role of digital technology in health systems strengthening.

From the onset of its design, implementation, monitoring and evaluation, the Afya-Tek program draws on multiple approaches combining surveys, in-depth interviews, focus group discussions and documentary reviews. We conducted surveys during monitoring and evaluation, and several rounds of supervision of the program activities. We also conducted in-depth interviews and focus group discussions with various stakeholders including healthcare actors (CHWs, ADDOs, health facility workers), beneficiaries (women at reproductive age and adolescents), Council/Regional Health Management Team (C/RHMT) and other government authorities. Data collection was done by a team of researchers and enumerators who were well trained on both quantitative and qualitative data collection methods and techniques. Our analysis forms part of a broader understanding of the Afya-Tek program which highlights potential grounds for strengthening the continuum of care at community level through digitally enabled systems. To arrive at a nuanced understanding of the Afya-Tek program, we also pay attention to the initial program set up, various lessons learnt and challenges encountered.

For the qualitative part, the audio data generated were transformed into texts and transferred into computer databases. Data was managed using the NVivo QSR (version 12 +). Analysis was undertaken by grouping relevant themes pertaining to research questions and program activities and research. On the other hand, quantitative data was analysed using the SPSS computerised software.

### Study setting

The Afya-Tek program is set in Kibaha district within the Pwani region of Tanzania. According to the latest census conducted in 2022, Kibaha district has an estimated total population of 388,727 [7]. Kibaha district is further subdivided into two councils: Kibaha Town Council (Kibaha TC) and Kibaha District Council (Kibaha DC). Each of these councils have their own local government authorities. Economic activities in Kibaha district include crop cultivation, livestock keeping, and small-scale businesses. Kibaha inhabitants include mixed ethnic groups and cultural diversities with Zaramo, Kwere, Mang’ati and Masai representing the majority. The two councils showcase the typical rural–urban disparity, in terms of access to and the utilisation of health-care services [24, 25], that can be found across Tanzania]. Being part of the Pwani region, Kibaha district residents represent strong coastal traditions that may favour early pregnancies [26, 27].

### Target populations

The target population of the Afya-Tek program includes the Afya-Tek digital system users and community beneficiaries. The users are CHWs, ADDO dispensers, and health facility workers (HFW). All eligible CHWs, ADDOs, and HFs within the two Kibaha councils were equipped with a smartphone, programmed with user-customised versions of the Afya-Tek system app. Beneficiary groups included all households within the catchment areas of both councils, with particular focus on: antenatal and postnatal women; children under age 5; and adolescents ages 11–19. Additionally, other key actors included: Village/Ward government leaders, Council/Regional Health Management Team (C/RHMT) members, as well as ADDO owners.

### Afya-Tek program and Afya-Tek system design

**Fig. 1:**
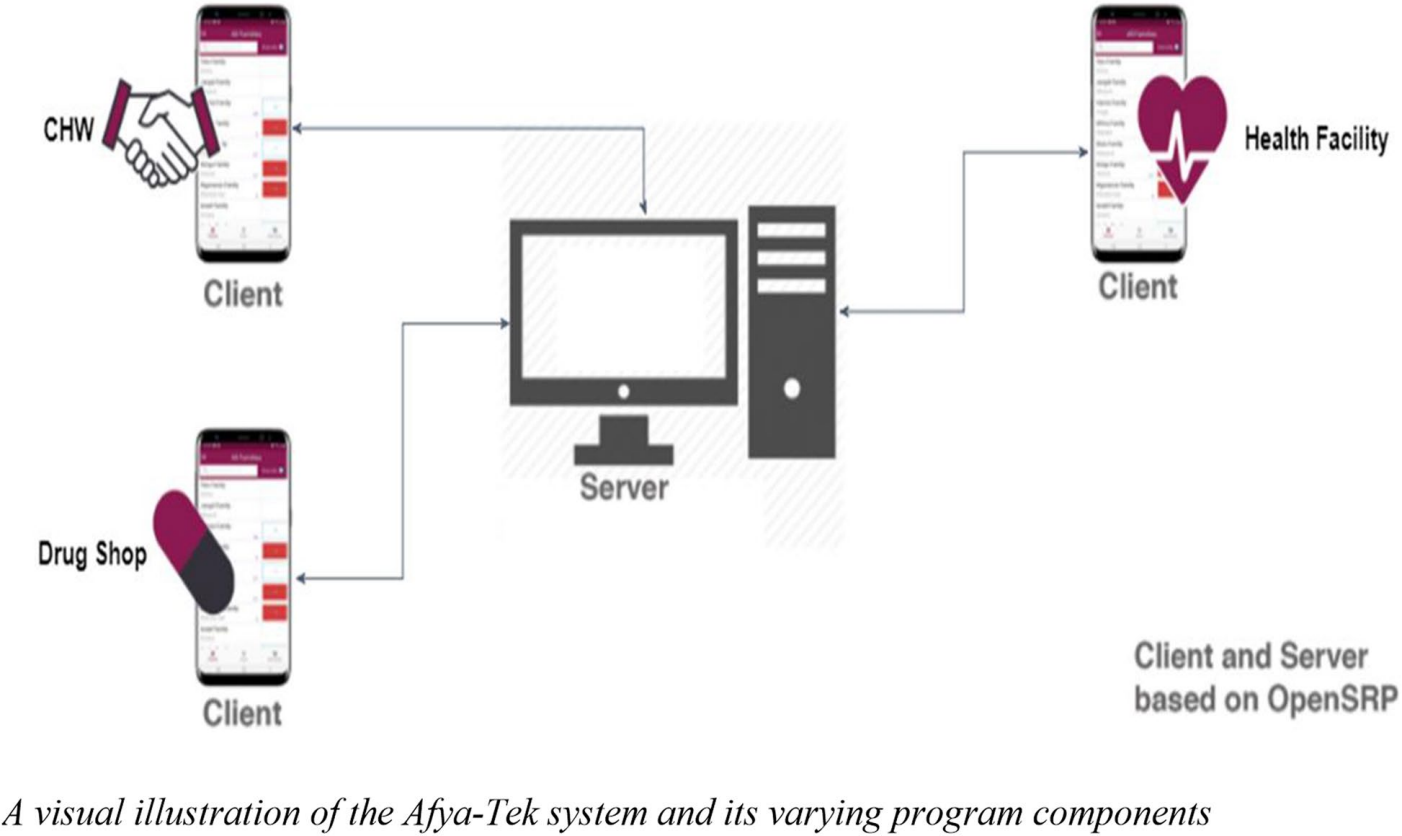
A visual illustration of the Afya-Tek system and its varying program components

Afya-Tek is a program and a digital system. The Afya-Tek program (see Fig. 1) coordinates care by linking together the three main actors of the Tanzanian primary health system, i.e. health service providers: namely CHWs, ADDOs, and HFs—through a digital referral system. Additionally, Afya-Tek registers community members, screens them for health danger signs, and facilitates digital referrals (issuing, following up, and closing) across health workers. This is achieved by equipping the three health worker groups with user-customised digital smartphone applications, tailored to the needs and responsibilities at each point of care. The applications are available in both Kiswahili and English languages. Finally, as part of its ongoing research and learning from the program, there is a specific focus on generating lessons and traversal learning using Realist methodologies.

## Participatory approaches to design

### Human-centred design

A primary facet in the conception and ongoing implementation of the Afya-Tek program was the use of a human-centred design approach. This is an approach that includes active participation of stakeholders (i.e., users of the solution and those affected by the solution) throughout program activities so that the solution fits the needs and preferences of stakeholders and is more likely to be adopted and sustained. Several steps were taken to ensure that the digital system is, and continues to be, closely aligned to local needs and resources, while simultaneously bringing together a wide array of local, national, and international experts. To that end, the Afya-Tek digital system was co-created and co-produced using community participatory approaches with all relevant stakeholders, such as the local communities, health systems actors, app-developers, researchers, and program implementers. Human-centred-design activities continued throughout the course of program activities so that the community’s and other relevant stakeholder needs were reflected as the program evolved. This helped to ensure that the digital system was created and adjusted to specifically meet the needs of those who would be using the technology, as well as be coordinated with the needs of the Tanzanian health system at large.

### Initial literature search and formative research

In July 2019, a thorough desk review of Tanzanian national health and technical documents was conducted. This review gave the Afya-Tek team valuable insights related to the current Tanzanian digital health strategy [21–23]. Combined with an in-depth exploration through the existing national initiatives and guidelines at the time [12, 28, 29], it allowed us to understand current best practices for CHWs, ADDOs, and HFs.

The desk review was part of a large-scale formative research across Kibaha TC and DC [26]. The purpose of this investigation was to develop an in-depth situational and contextual analysis, which explored existing barriers and facilitators to the continuum of care in the primary healthcare system. This included exploring current health seeking behaviours and practices of community members, as well as the motivations and challenges faced by the health service providers. More details on this study can be found in Haroun et al.’s publication [26].

The findings from these investigations informed the system design and allowed the Afya-Tek team to begin designing the Afya-Tek digital system as per the needs identified from the research.

### Government engagement

Government engagement has been key in program creation and implementation. The Afya-Tek team has been working closely with the MoH, PO-RALG (including district and village governments), and the Pharmacy Council. Each entity is highly influential and relevant to the Afya-Tek program in providing guidance and direction on how the program fits into Tanzania’s strategic plans. These included following all relevant guidelines and policies, as well as building the Afya-Tek system within Open Smart Register Platform (OpenSRP). This engagement and meeting were deemed crucial for Afya-Tek’s vision for program scale-up and sustainability within Tanzania.

### Digital system development

The Afya-Tek team began developing the digital system in November 2019 by following an agile development process, in which a set of functionalities were iteratively developed in three-week “sprints” or “cycles” of development. After each sprint, the Afya-Tek team tested the applications internally across 275 test cases to ensure that each new set of functionalities were working properly. Testing feedback was then triaged and worked back into development during the following sprint.

Once a “minimum viable product” (in which all essential functionalities were set up, but with the intention of further development) had been developed, user acceptance testing was conducted with users. All user feedback was then taken into consideration during subsequent development cycles.

### The developed Afya-Tek digital system

The Afya-Tek digital system consists of the CHW, ADDO and Health Facility OpenSRP applications or apps. The development of each app is described below.

### Afya-Tek CHW app

The mobile app developed for CHWs allows each CHW to register and manage all clients within the CHW’s village. At least one CHW has been assigned to each village/*mtaa* within the Kibaha district by the local government authority, and each CHW is responsible for registering all households and individuals within the catchment area.

**Fig. 2:**
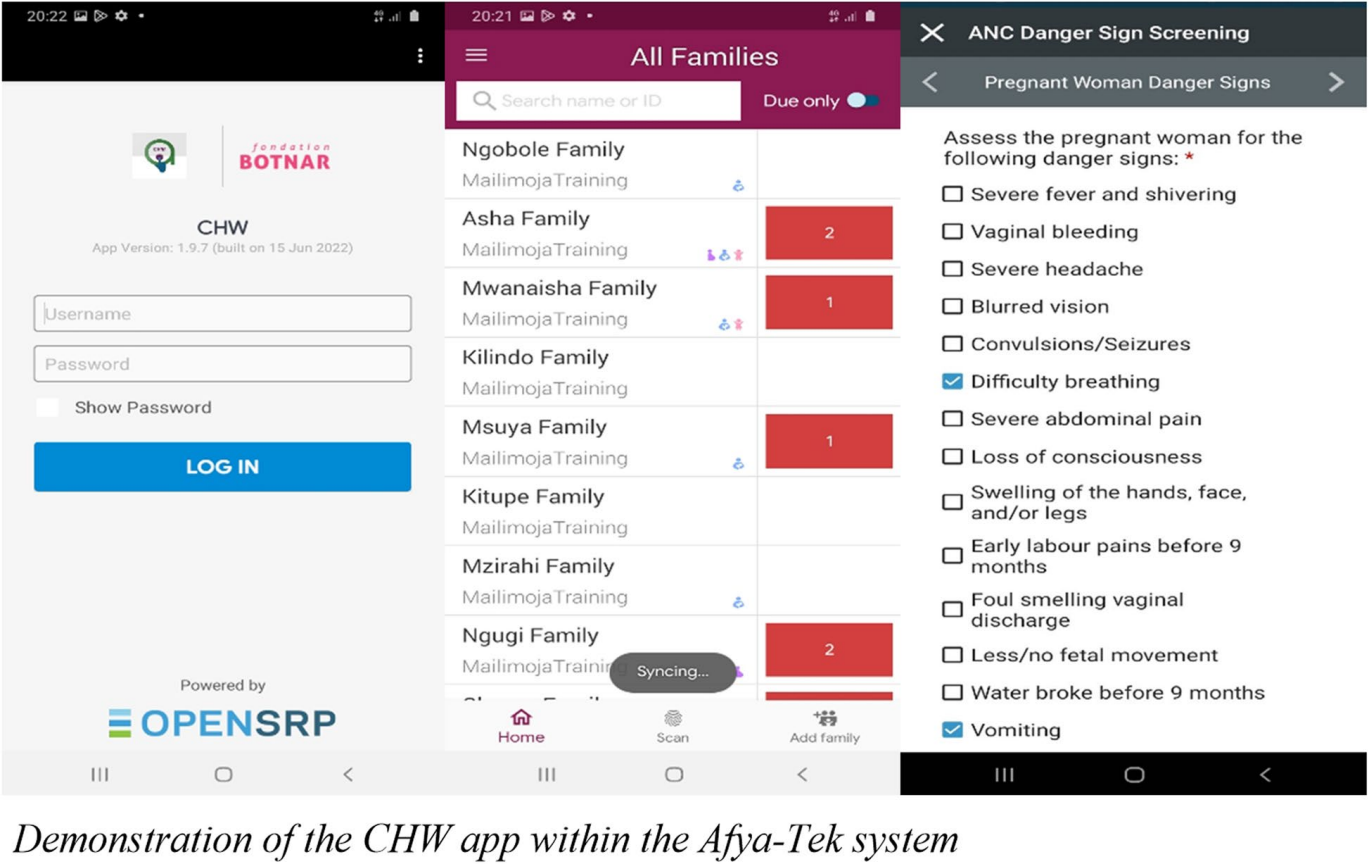
Demonstration of the CHW app within the Afya-Tek system

Once clients are registered in the Afya-Tek system, CHWs conduct home visits according to specific visit schedules in order to screen the client for danger signs, counsel the client or caretaker on age-appropriate health education and prevention topics. The whole screening process is guided by the CHW app (Fig. 2).

If the screening process uncovers a danger sign, the CHW is prompted by the app to either link clients to an ADDO, or refer the client to a health facility, depending on the severity of the issue. The Afya-Tek app also alerts CHWs of any follow-up visits that are due after referral completion. After the client completes the referral at the HF, the CHW is prompted by the system to then conduct a final follow-up visit to ensure the client received sufficient care and treatment.

The digital tool for CHWs was developed in accordance with all Tanzanian MoH guidelines [28, 29] for community care. These guidelines specify home visit schedules for new-borns, children under 5, and pregnant and postpartum women; symptoms to screen for during home visits; and which of these symptoms warrant a referral to the health facility.

### Afya-Tek ADDO app

**Fig. 3:**
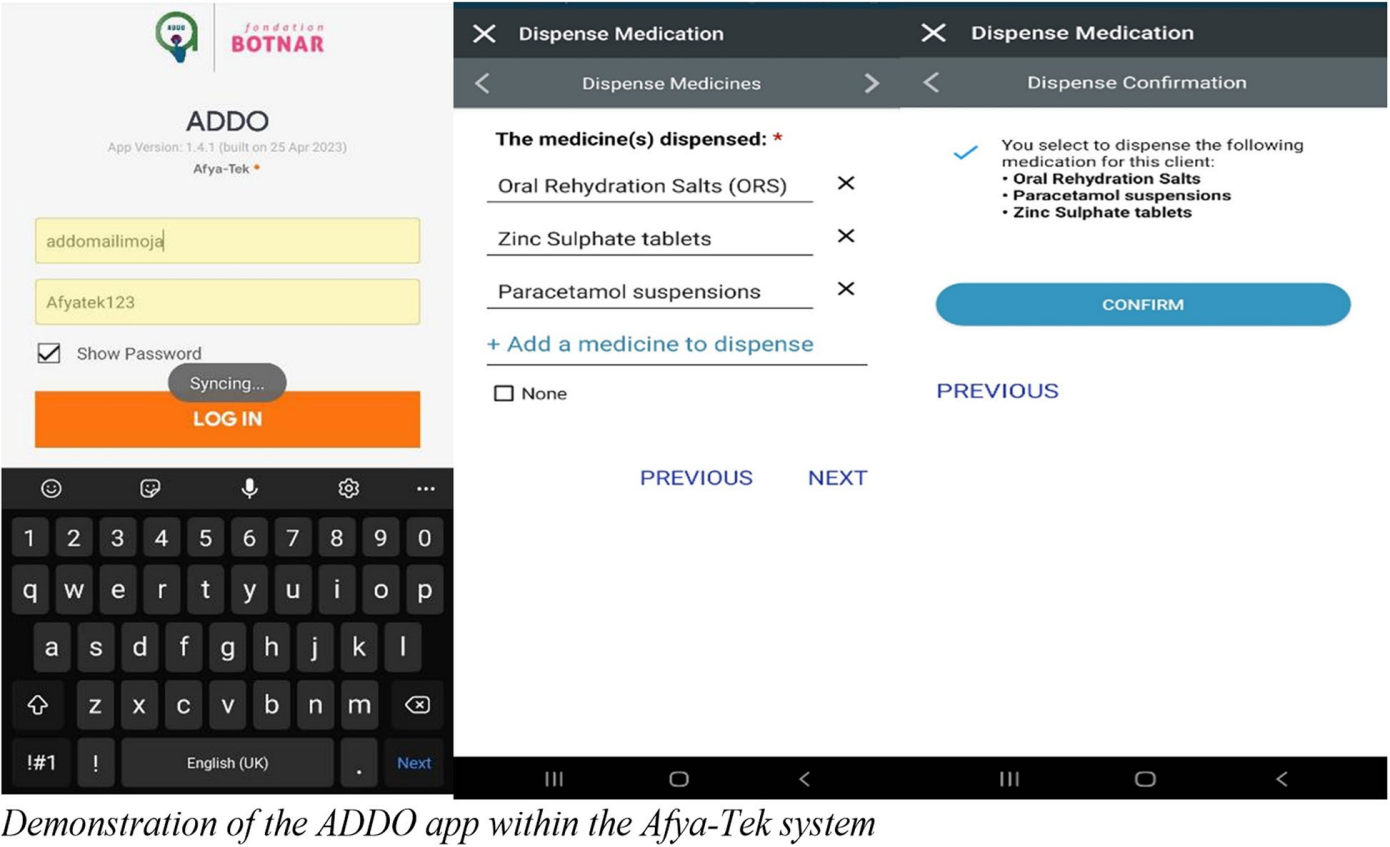
Demonstration of the ADDO app within the Afya-Tek system

The mobile app developed for ADDOs allows each ADDO to identify, screen, and treat each client that comes into the ADDO shop. After a thorough screening using the app, the ADDO dispenser can indicate back into the app what medications, if any, have been dispensed to the client. If the screening identifies any danger signs, the system prompts the dispenser to refer the client immediately to a health facility (Fig. 3).

The digital tool for ADDOs was developed in accordance with all Pharmacy Council, Tanzania Medicines and Medical Devices ADDO lists of medicines, and MoH guidelines for ADDO services [12, 28]. These guidelines specify symptoms to screen for during client visits and which of these symptoms warrant a referral to the health facility.

### Afya-Tek health facility app

**Fig. 4:**
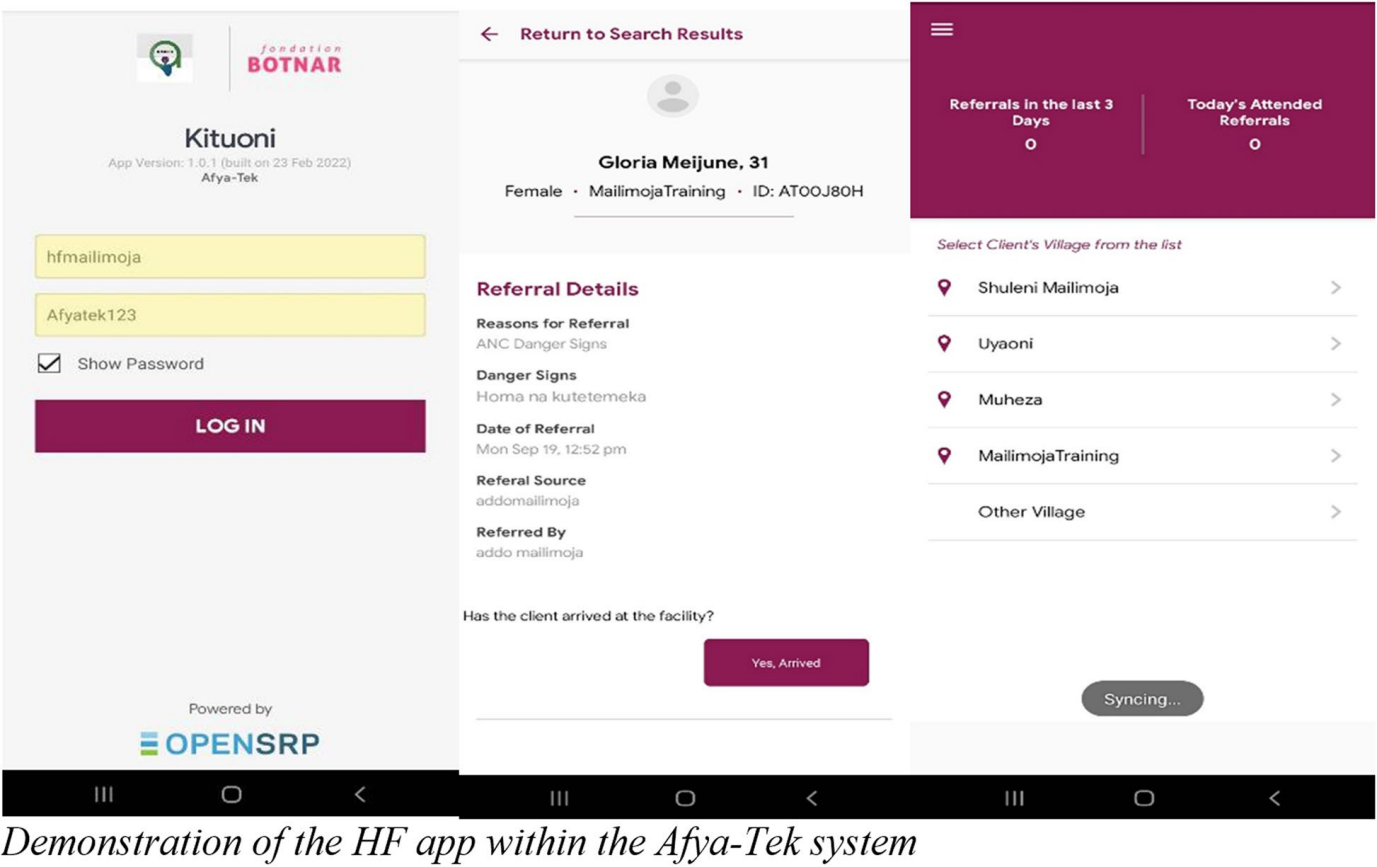
Demonstration of the HF app within the Afya-Tek system

The mobile app developed for HFs, gives health facility staff visibility of the Afya-Tek system’s referral information, including client symptoms, origin of the referral (whether from a CHW or ADDO), and time of the referral. Once a referred client (from a CHW or ADDO) reaches the health facility and checks in with a HFW, the HFW can confirm attendance via the Afya-Tek health facility app, and the status of that client’s referral on the CHW app changes to “Received” (Fig. 4). The CHW then knows that the client has completed the referral and is ready for a follow-up visit. In this way, Afya-Tek’s digitised process of referral allows for a complete circuit of referral and follow-up of clients, from home to facility and back to home (Fig. 4).

### App integration and coordinated care provision

Each of the three Afya-Tek health service provider apps is integrated with the others in order to strengthen the continuum of care at the primary level, reduce redundancy in assessments, and facilitate longitudinal tracking of client health (see Table 1). CHWs can link clients to ADDOs or refer to HFs; ADDOs can refer clients to HFs; and all clients completing referrals at HFs receive follow-up care from CHWs. In this way, the Afya-Tek system closes referral loops that are otherwise difficult to track and help provide sufficient support for clients.

**Table 1:**
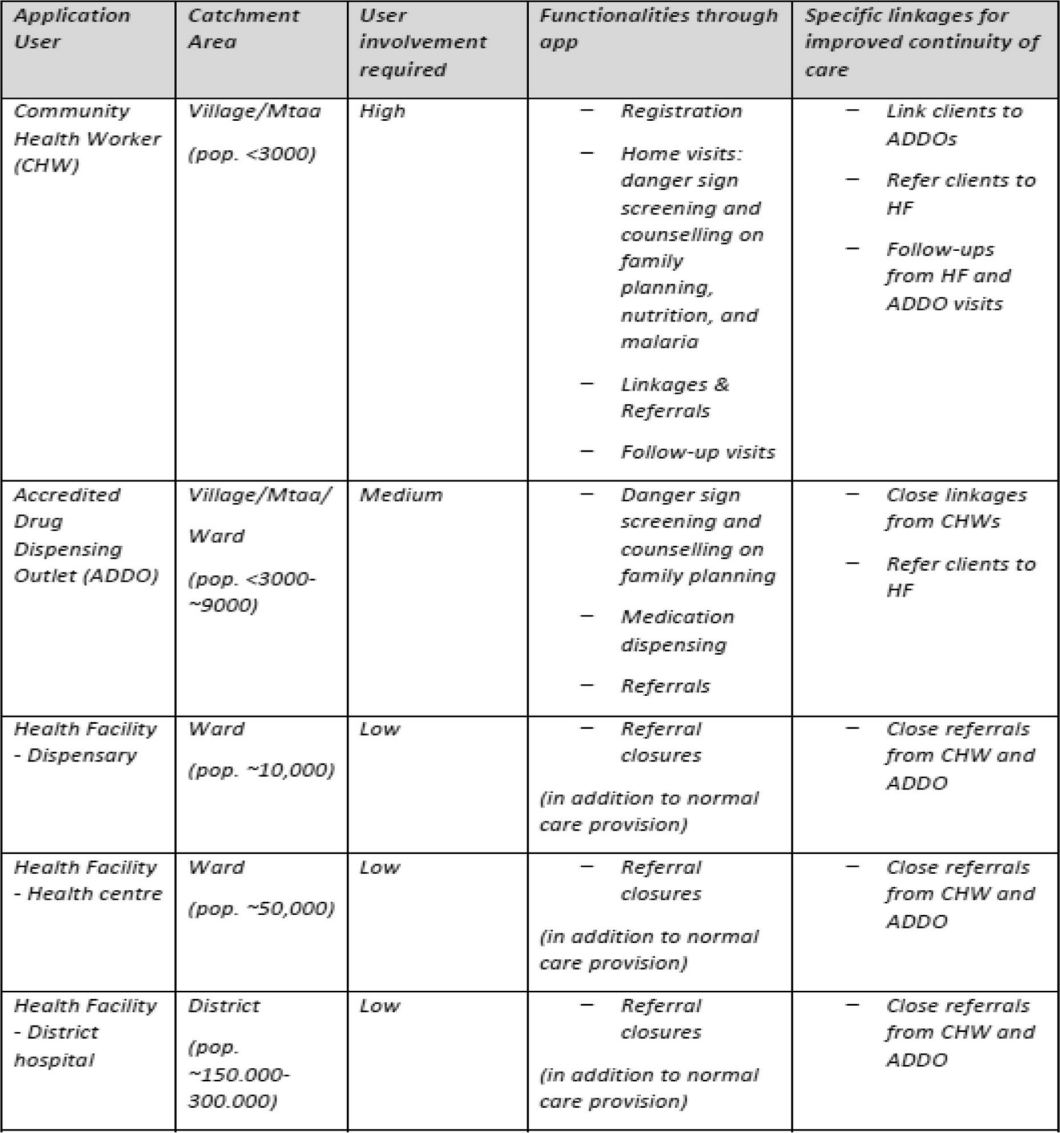
The created applications and their functionalities per user group

### App coordination and biometrics

Coordination of care across CHWs, ADDOs, and HFs is enabled by the “Afya-Tek ID” that is assigned to each client at registration. This unique identification number is used at each point of care to call up the client’s profile as he or she is identified. The client profile can be edited by CHWs, while the referral status can be updated at each point of care, meaning that health data captured by each service provider can be used to build a consistent, longitudinal client profile.

App coordination, longitudinal care and referral completion was originally supported by the biometric identification component of the Afya-Tek system. Biometric capture was done at the point of client registration by the CHWs, and at each future point of entry (whether at home with a CHW, at a drug shop with an ADDO dispenser, or at the health facility) clients were identified by thumb and forefinger scans. This was matched with the corresponding registered Afya-Tek ID and the matching client’s profile, and up-to-date referral information was retrieved and presented on the service provider’s app. However, this component was later removed as part of Afya-Tek’s iterative, responsive and adaptive system design.

### Sensitization, recruitment, and training

In February 2020, the Afya-Tek team led sensitization meeting sessions in the two Kibaha councils to inform stakeholders about the project, update them on its status, and seek their participation and collaboration. Sensitisation targeted community leaders, ADDO owners, C/RHMT members, and Council heads of various departments.

After sensitization, user training was done in two cohorts. Cohort I training (July 2020) involved only CHWs, who were trained exclusively on general phone usage, program objectives, consent protocols, biometric capture, and client registration. Cohort II training (October 2020) began once nearly 60% of the population of Kibaha had been registered by CHWs into the Afya-Tek system, and involved all users. Users were trained on home visits, referrals, follow-ups, medication dispensing, and referral closures, according to which component of the health system they represented and were required to use. The apps were released prior to user training in a staggered format in both July 2020 and September 2020.

CHWs received a performance-based monthly stipend (in line with government recommendations), mobile data and airtime bundles, along with all other health worker user groups, to facilitate reliable data syncing and communication.

## Monitoring and evaluation

The Afya-Tek program focused on a continuous and participatory approach to monitoring and evaluation (M&E) of the program. Multiple strands of work (outlined below) allowed for an iterative approach that has led to the development of a digital system that is more aligned with user and community needs.

A key learning and outcome of this agile and continuous M&E occurred in the fall of 2021, a year after implementation began, when it was acknowledged that the biometric component of unique identification was a recurrent barrier to implementation within this context. As such, the system was re-adapted without biometric identification processes to ensure smoother utilisation and more user- and client-friendly uptake of the Afya-Tek system.

### Continuous improvement and refinement of the system

The Afya-Tek team continued with OpenSRP system development to continuously and iteratively improve the system based on both internal testing and feedback from users. The Afya-Tek team’s strong testing and development processes, along with the follow-up activities outlined below, encouraged regular feedback gathering, prioritisation, and incorporation. By having open communication around the feedback, the Afya-Tek program was adaptable to the changes and information coming from users and remained flexible in planning the way forward for each iteration of the digital system.

### Continuous supervision and monitoring

Performance monitoring is an ongoing activity performed by the Afya-Tek team, CHW supervisors and CHMT. The team monitors the performance of the end users and provides on-site support (technical or advisory), guidance and instruction with respect to the Afya-Tek system competencies, and supportive supervision by documenting challenges found on the field and identifying areas for improvement.

Various monitoring platforms utilised by the program include a) remote support through dashboard monitoring, communication via WhatsApp groups, and by conducting follow up calls to users, and b) physical monitoring through supervision visits where the Afya-Tek team and CHMTs observe user interaction with clients and provide on-site support where needed. (More information can be found in Appendix 1).

### Dashboards, data sharing and communication across stakeholders

A key component of the M&E process was to create supervisory dashboards at the council levels to evaluate system activity and track key indicators that could assess program progress and/or user performance. All dashboards were customised based on the user (supervisors, CHMTs, and Afya-Tek program teams) and on the user device type (e.g., the supervisor dashboard was configured to be viewed on a phone, whereas the CHMT dashboard was configured for desktop view).

The dashboards have been critical for program M&E in order to increase data transparency and accessibility across partners and serve as a platform to conduct M&E analyses with large datasets. Being actively part of this M&E process has been instrumental in particular for consortium partners and for CHMTs in decision-making and resource planning. Any feedback or recommendations based on discussions have been continually used to strengthen dashboards and program activities. Examples of data utilisation metrics include monitoring the frequency of dashboard access, tracking the types of analyses conducted, and assessing changes in programmatic activities driven by data insights.

### Realist monitoring and evaluation

The Afya-Tek program has at its core a major learning objective: To generate transversal learnings, that are based on context-specific understandings of how digital innovation within the continuum of care at primary care level can lead to responsive, people-centred healthcare which improves the maternal, child and adolescent health outcomes in coastal Tanzania.

Realist Evaluation (RE) is a structured, yet flexible, research approach that combines quantitative and qualitative research methodologies to monitor, intervene, and evaluate a program's impact [30]. The basic premise under which it operates is the observation, analysis, and explanation of how*, why, for whom, and under what circumstances* an intervention does or does not succeed [31].

Realist evaluation is particularly useful for evaluating new initiatives as it allows one to fully understand and delineate their complexities and see how best to adapt the intervention within new contexts, thereby informing potential program scale up. As such, multiple strands of mixed-methods research were conducted throughout the program implementation, with ongoing programmatic interventions and outcomes iteratively informing each strand of research. The key RE study components within Afya-Tek are highlighted in Box 1. (See Appendix 2 for more information).

## Preliminary findings & analysis

The Afya-Tek full system deployment began in November 2020. With 2.5 years of implementation thus far, preliminary findings of the program highlight that current service provision through the app is coordinated across approximately 400 health providers (240 CHWs, 110 ADDOs, and 53 HFs) within Kibaha (Dillip et al., The Afya-Tek Final Report, April 2019-June 2023, Unpublished Report). Table 2 provides initial data and key achievements of the Afya-Tek program since its deployment into Kibaha district (July 2020—June 2023).

**Table 2:**
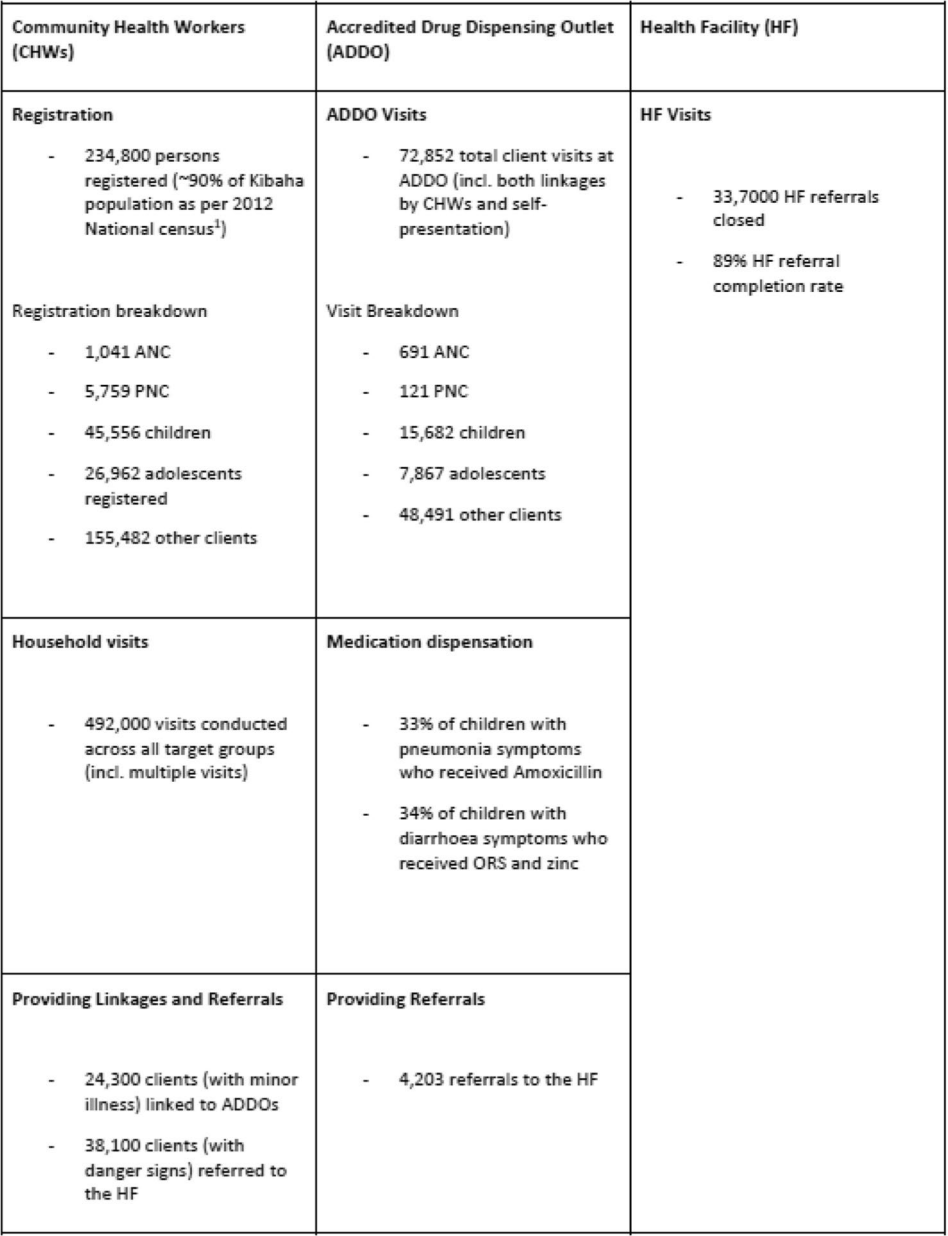
Initial data and key achievements of the Afya-Tek program since its deployment into Kibaha district (July 2020 - June 2023)

^1^2022 National Census disaggregated data available since 2023 projects Kibaha population at 388,727 bringing down the registration status to 60%

Key highlights from this data include: CHWs being instrumental in counselling clients during household visits regarding malaria prevention, family planning and nutrition. As indicated in Table 2, a total of 492,000 visits conducted across all target groups (incl. multiple visits) provides an opportunity for CHWs to conduct various counselling sessions. The contribution of the ADDO as a private health sector actor within the continuum of care is quite evident particularly with children and adolescents; 33% of children with pneumonia symptoms received Amoxicillin, 34% of children with diarrhoea symptoms received ORS and zinc, and a total of 7,867 adolescents have been attended at the ADDO level. With the 89% linkage and referral completion rate from CHWs to ADDOs and HFs, is a positive indicator of a stronger coordination system (Dillip et al., The Afya-Tek Final Report, April 2019-June 2023, Unpublished Report).

Additionally, the Afya-Tek system provides an easy-to-use supervisory mechanism for the CHW supervisors to easily monitor performance of the CHWs. Through this, CHW supervisors are able to assess referral issues by CHWs, source of referral (CHW or ADDO), danger signs screened and accuracy of referrals made. As well as for the CHMTs, they are able to digitally monitor and follow progress within their councils, which has subsequently aided them in resource management and planning. Further details of Afya-Tek’s results and outcomes will be shared in upcoming publications.

In the course of implementing the Afya-Tek program, several lessons learnt were documented, these include; value-based alignment, motivation and perception around use of digital tools, preference of adolescent seeking care at ADDOs, the value of Government, community and multi-stakeholder engagement in program implementation and the relevance of private sector inclusion into digital public service provision (see Table 3 for key lessons learnt).

**Table 3:**
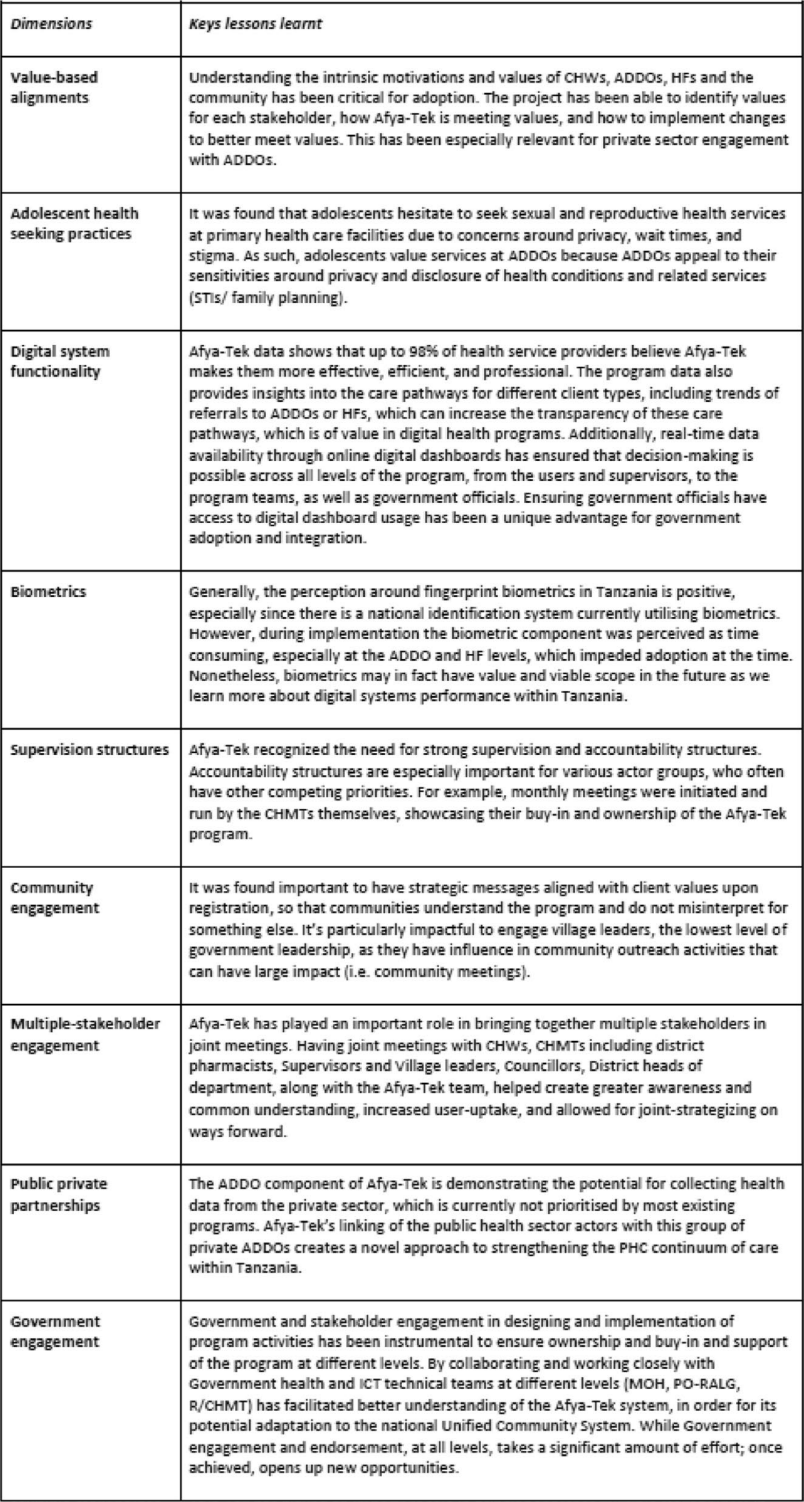
Key lessons learnt through the Afya-Tek program

A multi-user program such as Afya-Tek is complex, and requires significant efforts and time, as well as regular monitoring and problem-solving to overcome challenges. Being a complex program bringing together public and private health sector actors, Afya-Tek naturally experienced various implementation challenges. As ongoing challenges were encountered, due the responsive and agile nature of the program, Afya-Tek ensured prompt mitigation and problem solving. These challenges can be categorised as either being contextual or technical challenges. As challenges differed based on location and target-population, the solutions thus needed to be context- and actor-specific. As with all in-field challenges, solutions were continuously generated and rolled-out in an iterative design. Table 4 highlights some of the challenges faced by the program and identified solutions thus far. These covered internet connectivity, incentivization model, lower system utilisation at ADDO level, placement of mobile devices at health facilities, acceptance of intervention by communities and COVID-19 (Table 4).

**Table 4:**
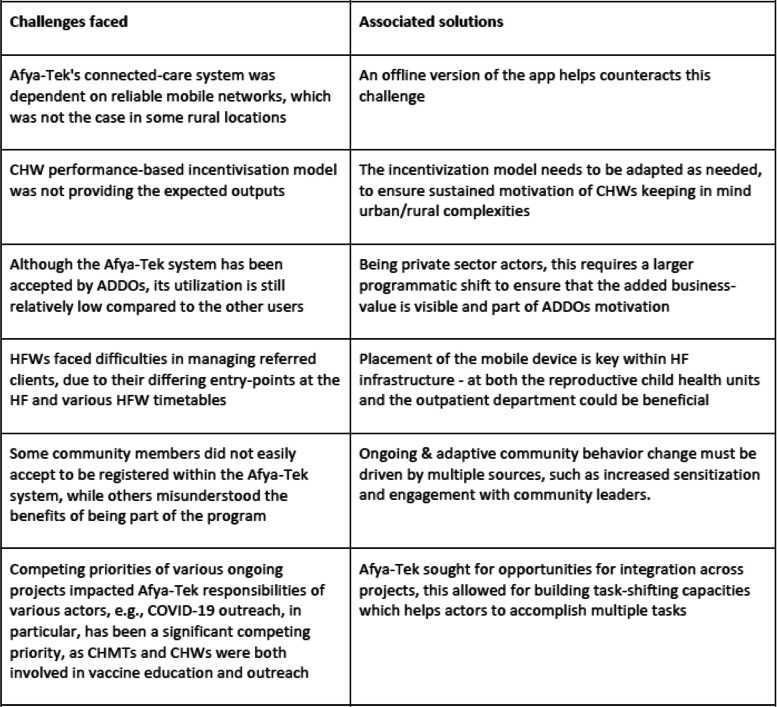
Specific challenges and associated solutions as encountered in the Afya-Tek program

## Discussion & conclusion

The Afya-Tek program is the first digital health system in Tanzania to demonstrate the link among CHWs, private sector ADDOs, and HFs for a strengthened continuum of care. It purports to create and effectively implement an innovative digital health intervention that ensures the provision of smooth, coordinated care among primary healthcare system actors for communities in the Pwani region of Tanzania. Afya-Tek thus demonstrates a proof-of-concept model that addresses the problem of coordination of care.

As an illustration of the PPP model in Tanzania, ADDOs are proving to be an important entity for capturing clients with illnesses at the community level, managing them and providing referrals to HFs. Strengthening the use of these private sector platforms also offers unique opportunities for reaching adolescents and providing them with person-centred and confidential services with easier access to contraceptives and condoms. Going forward, the program intends to strengthen the adolescent health component by incorporating adolescent youth clubs to ensure greater engagement of the youth.

Afya-Tek also provides a potential platform to test and incorporate other disease conditions. For instance, this can include exploring the addition of other national high priority health areas, such as NCDs (e.g., diabetes, hypertension) and nutrition, and other infectious diseases like tuberculosis and HIV. The platform can be used to ensure appropriate management of these conditions and access to services and medications across the continuum of care.

With the Afya-Tek system being built on the OpenSRP platform, it has a broad endorsement from the Government of Tanzania. This allows for interoperability and integration within national digital health systems such as the Unified Community System (UCS), Government of Tanzania-Hospital Management Information System (GoT-HoMIS), and District Health Information System 2 (DHIS2). The potential for continued data-use from the Afya-Tek system is high, and thus next steps include continuing on-going collaboration and engagement with health and ICT technical teams at various levels of Government (MOH, PO-RALG, Pharmacy Council, R/CHMT) for eventual integration and adaptation into the National digital community system.

By and large, the findings reflected in this article are consistent with ongoing discussions in the digital health literature, both nationally and within sub-Saharan Africa [32, 33]. Despite the growth of digital technology in recent years, Tanzania, like other LMICs, still faces a problem of the digital divide. In general, while urban settings by and large have access to the benefits of emerging digital technologies, the majority of rural areas still experience low access [34]. In regards to scaling up and sustainability in the LMICs, there is a consensus that the success of digital health intervention and subsequent scaling up of the same require several interrelated pillars [35]. These include, but are not limited to, the intrinsic features of the programme; stakeholder engagement; simplicity, interoperability and adaptability; alignment with policy environments; and adaptable extrinsic ecosystems. Additionally, it remains crucial to understand the role of artificial intelligence decision support tools, as our consortium partner Inspired Ideas has been exploring [36].

Thus, there is much scope and potential for a program like Afya-Tek’s, which bridges this digital divide, to have a significant impact on health outcomes in the long run. Through such a digitally coordinated system, the Tanzanian PHC system would be able to digitally create efficiency and communication in referral systems across public and private primary healthcare providers. Discussions on long-term sustainability allow for possibilities to scale across other settings within Tanzania, alongside generating transversal learnings to utilise in other LMICs contexts. If continuity of care and follow-up could be made efficient by digital innovations such as the Afya-Tek system, the quality and coordination of primary care would be improved especially in resource constrained settings; thereby allowing focus back to ensuring equitable, quality, person-centred care for all.

### Limitations of the program

Despite the initial achievement highlighted, limitations or challenges encountered during program implementation may impact program effectiveness. Inclusion of ADDOs into formal digital public service delivery may pose a challenge in early adoption and acceptance of digital health interventions. Apart from ADDOs being service providers, they are also interested in profit making, with this regard any intervention at the outlets should also ensure these entities benefit from medicine and commodities sales. Health entrepreneur opportunities may be relevant to be explored in future to sustainability of initiatives like Afya-Tek. On the other hand, given the ongoing trend of digital interventions in low resource countries, the question of interoperability with Government systems remains critical towards realisation of effectiveness of the program. However, working closer to the government through its ministries, MoH and PO-RALG, there has been a move towards integrating the Afya-Tek system into the National Unified Community System (UCS). While this has potential for scalability of the Afya-Tek system countrywide, it also brings a great value of integrating the system into the national agenda and foster sustainability and incorporation of other disease conditions.

### Areas for further research

Based on the initial findings of the Afya-Tek program, this study recommends the following areas for further investigation. First, exploring the program's long-term sustainability beyond the initial implementation phase. Secondly, evaluating the cost-effectiveness of digital health interventions in low-resource settings. Lastly, investigations that seek to understand the mechanisms underlying successful referral coordination, exploring the role of community engagement in sustaining program outcomes, and investigating the scalability of similar digital health interventions in different cultural contexts are key to a nuanced understanding of the digital health programs similar to Afya-Tek.

### Supplementary Information

Supplementary Material 1.

Supplementary Material 2.

Supplementary Material 3.
