# Supplementary material for: Using digital technology as a platform to strengthen the continuum of care at community level for maternal, child and adolescent health in Tanzania: introducing the Afya-Tek program
Source: BMC Health Serv Res. 2024 Jul 30;24:865. doi: 10.1186/s12913-024-11302-7 (PMC11290070; doi:10.1186/s12913-024-11302-7)
Supplement: Supplementary file 1 — Supplementary Material 1. [file 12913_2024_11302_MOESM1_ESM.pdf]

Appendix 1

Supervision Structures

The Afya-Tek team set up strong supervision structures so that CHWs, ADDOs, and health facility staff have the support that they need as they are using the system to ensure high adoption and skills. An ongoing follow-up hierarchy was established using both existing and additional structures operating within the CHW, ADDO, and health facility systems. In effect, firstly direct communication between the Afya-Tek team and CHMT was established through weekly virtual meetings; while secondly, direct communication between Afya-Tek, CHMT and CHW/ADDO through social media (WhatsApp groups). The following diagram shows the reporting hierarchy for questions, feedback, and issues.

CHW **ADDO** **Health Facility**

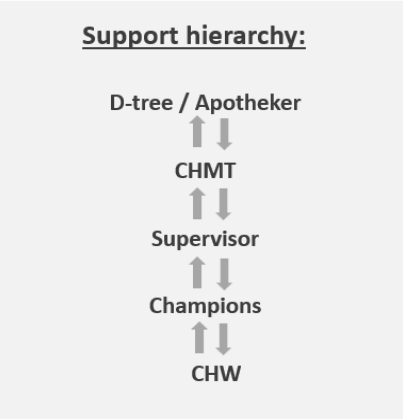

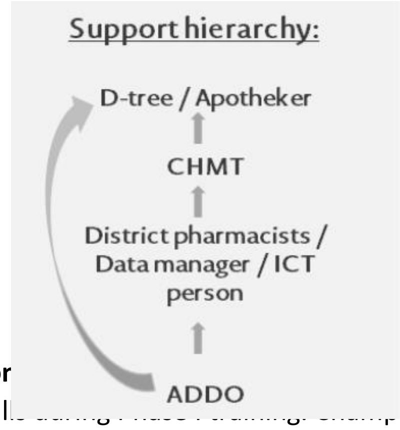

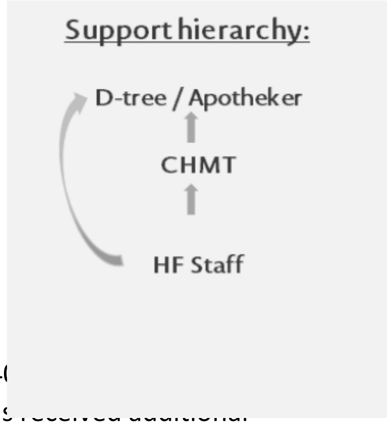

- [illegible]pior[illegible] 24[illegible] skil[illegible] ons[illegible] training to assist with any technical challenges experienced by fellow CHWs during the first few months of implementation. Champions have become an important link among CHWs, the local government authorities, CHW supervisors, and directly to CHMTs.

- **CHW supervisors** are a group selected by district authorities to monitor, support, and report to the district authorities on CHW activities. For the Afya-Tek program, a total of 60 supervisors were selected from among health facility staff throughout the Kibaha district to provide support either directly to CHWs or through CHW Champions. Supervisors received Afya-Tek mobile phones, which have helped them to troubleshoot issues and to monitor progress of CHW activities. They have also received a monthly stipend to support them when travelling to periodically supervise CHWs in person, as well as airtime and data bundles to facilitate reliable communication.

- The **Council Health Management Team (CHMT) members** monitor project progress and address both administrative and technological challenges reported by CHW Champions, supervisors, and sometimes directly by CHWs. They also monitor progress and challenges from ADDO dispensers and HFs.

- The **Afya-Tek team** addresses technological, administrative, and programmatic challenges reported by CHMTs and supervisors.
