# Supplementary material for: Using digital technology as a platform to strengthen the continuum of care at community level for maternal, child and adolescent health in Tanzania: introducing the Afya-Tek program
Source: BMC Health Serv Res. 2024 Jul 30;24:865. doi: 10.1186/s12913-024-11302-7 (PMC11290070; doi:10.1186/s12913-024-11302-7)
Supplement: Supplementary file 2 — Supplementary Material 2. [file 12913_2024_11302_MOESM2_ESM.pdf]

Appendix 2

Table a: Quantitative data collection details

**Quantitative survey**<br>**(time of data collection)**	**CHWs**	**ADDOs**	**HFWs**	***Total no. of participants***
**Round 1 : CHW (Aug 2020)**	**197**	**-**	**-**	***197***
**Round 2: System users (Nov 2020)**	**181**	**72**	**34**	***287***
**Round 3: System users follow-up (Mar 2021)**	**120**	**100**	**48**	***268***
**Round 4: System users follow-up (May 2021)**	**228**	**113**	**47**	***388***
Total no. of responses	***726***	***285***	***129***	***1140***

Table b: Qualitative data collection details

**Qualitative study**<br>**(time of data collection)**	**Setting**	**IDIs**	**FGDs**
**Study A : Multi-stakeholder community perceptions (Dec 2020)**	**Kibaha TC**	**17**	**6**
	**Kibaha DC**	**15**	**6**
**Study B: Multi-stakeholder community follow-up (Jun 2021)**	**Kibaha TC**	**17**	**1**
	**Kibaha DC**	**16**	**1**
**Study C-a: Consortium partners (Jun-Aug 2021)**	**Dar es Salaam**<br>**Online**	**7**	**3**

**Study C-b: Key informants (Oct-Nov 2021)**	**Dodoma**<br>**Dar es Salaam**<br>**Kibaha TC**<br>**Kibaha DC**	**9**	**0**
**Study D: Multi-stakeholder community follow-up (Aug-Nov 2022+Jan 2023)**	**Kibaha TC**	**50**	**3**
	**Kibaha DC**	**50**	**3**
TOTAL no. per method		***181***	***23***
