# Supplementary material for: Using digital technology as a platform to strengthen the continuum of care at community level for maternal, child and adolescent health in Tanzania: introducing the Afya-Tek program
Source: BMC Health Serv Res. 2024 Jul 30;24:865. doi: 10.1186/s12913-024-11302-7 (PMC11290070; doi:10.1186/s12913-024-11302-7)
Supplement: Supplementary file 3 — Supplementary Material 3. [file 12913_2024_11302_MOESM3_ESM.pdf]

Box 1: Key RE study components within Afya-Tek

1. IRPT Workshop 1: Initial development workshop	(Jun 2020)
2. Quantitative Survey Round 1: CHW	(Aug 2020)
3. Quantitative Survey Round 2: HW	(Nov 2020)
4. Qualitative Study A: Multi-stakeholder perceptions	(Dec 2020)
5. Quantitative Survey Round 3: HW follow-up	(Mar 2021)
6. Quantitative Survey Round 4: HW follow-up	(May 2021)
7. Qualitative Study B: Multi-stakeholder follow-up	(Jun 2021)
8. Qualitative Study C-a: Consortium partners	(Jun-Aug 2021)
9. IRPT Workshop 2: Refinement workshop	(Sep 2021)
10. Qualitative Study C-b: Key informants	(Oct-Nov 2021)
11. Qualitative Study D: Multi-stakeholder follow-up	(Aug-Nov 2022 + Jan 2023)
